# Bacterial endocarditis presenting in an adult patient with undiagnosed double‐chambered right ventricle

**DOI:** 10.1002/ccr3.2107

**Published:** 2019-03-25

**Authors:** Neil Salil Bhambi, Abhishek Shah, Patrick Sarte, David Robert Laughrun

**Affiliations:** ^1^ Department of Internal Medicine Keck School of Medicine of the University of Southern California Los Angeles California; ^2^ Department of Cardiovascular Medicine Keck School of Medicine of the University of Southern California Los Angeles California

**Keywords:** congenital heart disease, double‐chambered right ventricle, endocarditis, Tetralogy of Fallot

## Abstract

Double‐chambered right ventricle (DCRV) is a rare congenital heart defect often associated with ventricular septal defect and pulmonary stenosis. Cardiac catheterization or magnetic resonance imaging can differentiate between DCRV and Tetralogy of Fallot when echocardiogram is inconclusive. Patients are at an increased risk for bacterial endocarditis.

## 1 BACKGROUND

A double‐chambered right ventricle (DCRV) is a rare congenital heart defect characterized by a right ventricle (RV) that is separated into a proximal high‐pressure chamber and a distal low‐pressure chamber by a muscle band within the right ventricular outflow tract (RVOT). It is seen in 0.5%‐2.0% of all congenital heart disease (CHD) patients.^1^ Although most patients present with DCRV by childhood and adolescence, some will present during adulthood.^1^, ^2^ Infective endocarditis is a complication of CHD and can be seen in patients with and without surgical repair.^3^ We present a 26‐year‐old patient with an undiagnosed DCRV and ventricular septal defect (VSD) presenting with bacterial endocarditis.

## 2 CASE PRESENTATION

A 26‐year‐old gentleman with a past medical history of hypertension, presented to our hospital with persistent fevers for 2 weeks. He was in his usual state of health when he began developing daily fevers associated with proximal shoulder and thigh weakness. His fevers were refractory to antipyretics. He denied any other associated symptoms including rashes, arthralgias, myalgias, headache, nuchal rigidity, cough, abdominal pain, nausea, diarrhea, or dysuria. He had not recently travelled outside of the United States and denied any sick contacts. His last sexual encounter was 1 year prior to presentation. He denied genital lesions or discharge. He reported no history of drug abuse. He was born in the Philippines and immigrated to the United States 9 years prior to presentation. At birth, the patient was told he had a hole in his heart that would spontaneously close by adolescence. He recalled “turning blue” while crying as a child, but had since denied cyanotic spells since childhood. As an adult, he could walk several blocks and climb flights of stairs without difficulty. However, he reported dyspnea with jogging and running, which he had attributed to deconditioning. He was unaware of any significant family history.

**Figure 1:**
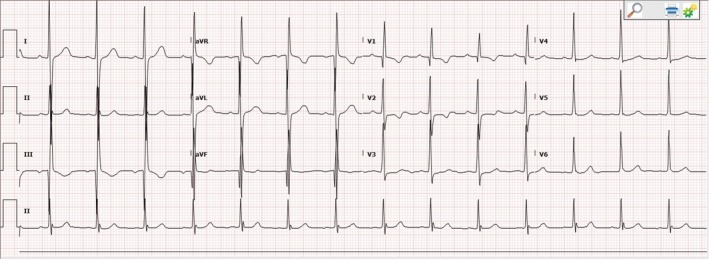
Electrocardiogram showing normal sinus rhythm, normal axis, increased voltage, prominent R waves in V1‐V6, and anterior ST segment and T wave changes

Upon arrival to the emergency department, he was afebrile and hemodynamically stable. The physical exam was notable for left, anterior, nontender, mobile cervical adenopathy and a III/VI holosystolic murmur heard loudest at the 3rd left intercostal space. Initial labs were notable for a mild leukocytosis and elevated erythrocyte sedimentation rate and c‐reactive protein. Electrocardiogram (EKG) showed increased voltage, prominent R waves in the precordial leads, and nonspecific ST segment and T wave changes (Figure 1). Chest X‐ray showed cardiomegaly and a chest computed tomography showed multiple pulmonary nodules in bilateral lung fields.

**Figure 2:**
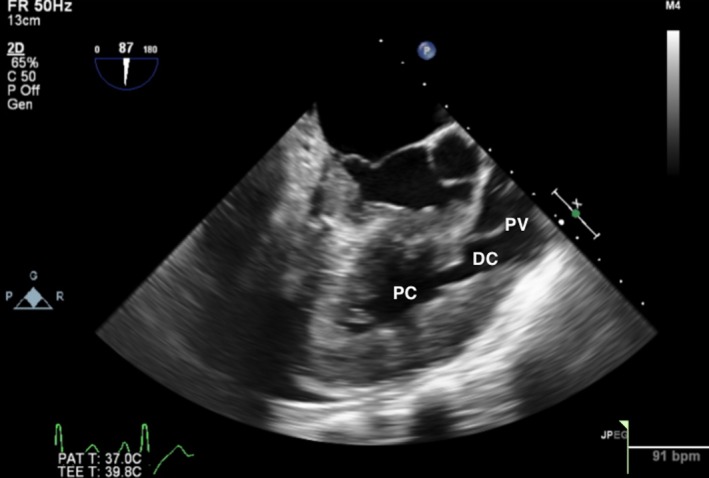
Transesophageal echocardiogram showing the right ventricle with a proximal chamber (PC) and distal chamber (DC) separated by a muscular band

**Figure 3:**
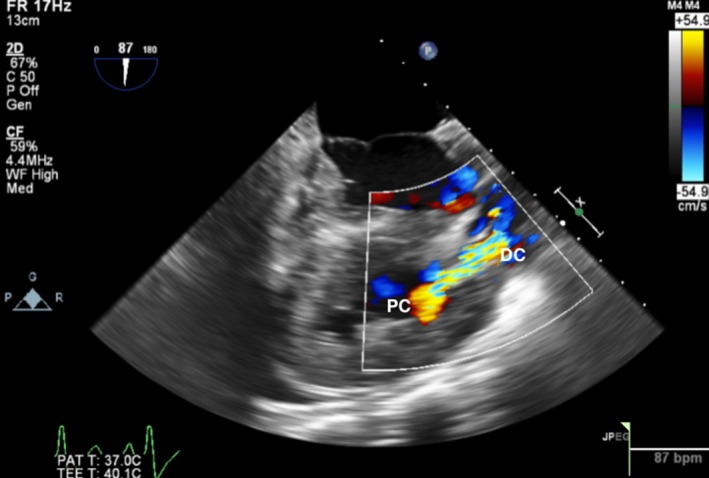
Transesophageal echocardiogram with Doppler signal showing flow acceleration across the proximal chamber (PC) and distal chamber (DC)

**Figure 4:**
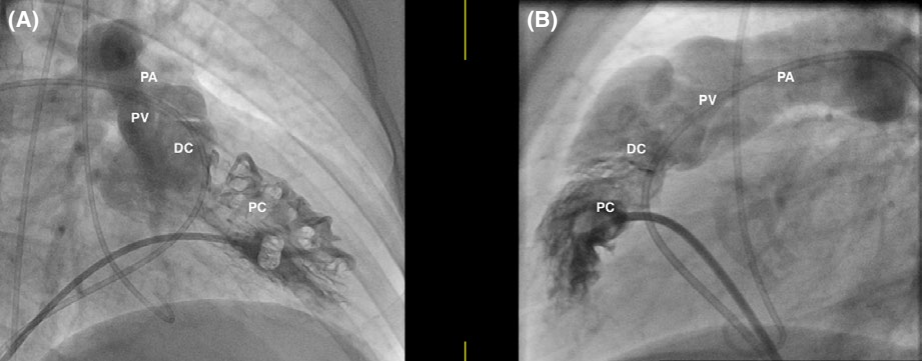
Cardiac catheterization showing trabeculated proximal chamber (PC), smooth DC (distal chamber), pulmonary valve (PV), pulmonary artery (PA)

**Figure 5:**
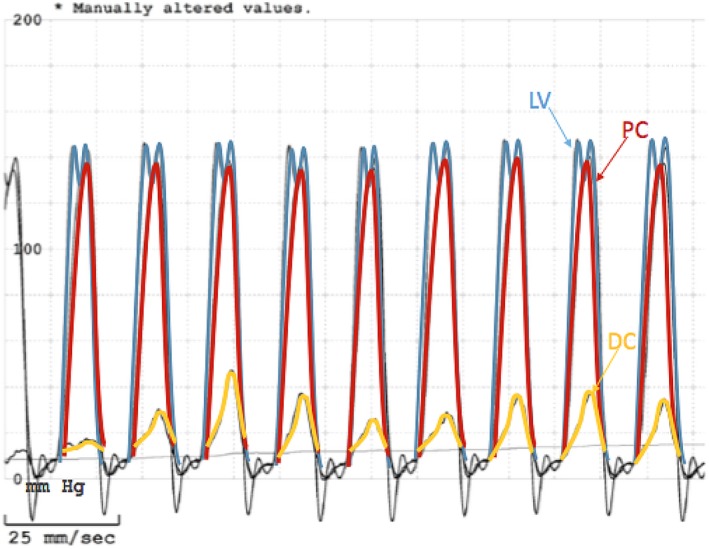
Cardiac catheterization hemodynamic data showing pressures of the left ventricle (LV), proximal chamber (PC), and distal chamber (DC). Note the significant pressure gradient between the PC and DC

**Figure 6:**
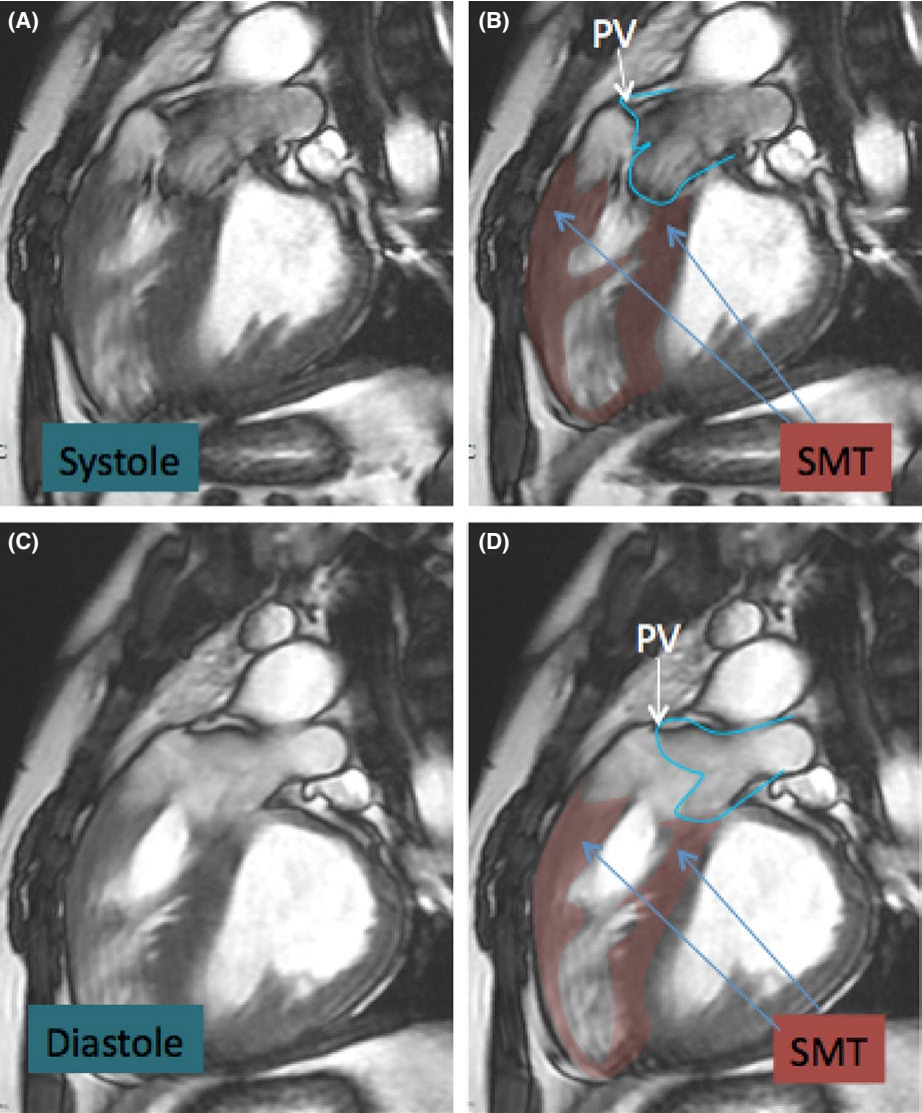
Long‐axis view of cardiac magnetic resonance imaging (MRI). Panel A and B showing right ventricular outflow tract (RVOT) subpulmonic stenosis during systole with pulmonary valve (PV) and septomarginal trabeculation (SMT). Panel C and D showing RVOT subpulmonic stenosis during diastole

A transthoracic echocardiogram (TTE) was obtained and showed moderate‐to‐severe right ventricular hypertrophy (RVH), RVOT, and a VSD consistent with Tetralogy of Fallot (TOF), although no evidence of an overriding aorta. He subsequently underwent a transesophageal echocardiogram (TEE) that again showed RVH and a VSD, but also noted a muscular band within the RV (Figures 2 and 3). Blood cultures grew aggregatibacter aphrophilus. He was diagnosed with endocarditis by Duke's criteria: one major (positive blood culture with HACEK organism), and three minor (fever, predisposing cardiac lesion, pulmonary emboli). He was discharged with a 4‐week course of intravenous Ceftriaxone following the last negative blood cultures. After discharge, the patient was further evaluated to differentiate between TOF and DCRV. A cardiac catheterization showed an anomalous band dividing the RV into two chambers, with a peak‐to‐peak gradient of 115 mm Hg between proximal and distal RV consistent with DCRV (Figures 4 and 5). Additionally, the patient underwent a cardiac magnetic resonance imaging (MRI) showing a D‐shaped left ventricle and RVOT with subpulmonic stenosis (Figure 6). He was referred to cardiothoracic surgery for RV muscle band myomectomy and patch closure of VSD.

## 3 DISCUSSION

This adult patient with undiagnosed, unrepaired DCRV and ventricular septal defect presented with bacterial endocarditis. DCRV is a rare form of CHD where the RV is divided into proximal high‐pressured and distal low‐pressured chambers. There are two classifications of DCRV described by Galiuto et al. In Type I, an anomalous muscular band crosses the RV cavity causing obstruction. Type II DCRV is characterized by hypertrophy of the parietal muscle and crista supraventricularis.^4^ Although DCRV can present as an isolated pathology, it is predominantly seen with associated congenital lesions. Up to 90% of cases are associated with VSD—other lesions seen include pulmonary stenosis—TOF and double outlet RV.^1^, ^5^ Due to the strong association with DCRV and additional congenital defects, some believe DCRV is an acquired lesion resulting from increased intraventricular pressure changes causing hypertrophy of the trabeculae.^1^, ^5^ Moustafa et al^6^ describe a case series of five patients with a prior history of VSD, who subsequently developed DCRV later in life, again suggesting DCRV may be an acquired condition.

Patients can present with variable symptoms depending on the degree of obstruction and associated lesions. However, most patients will have exertional dyspnea, syncope, or angina.^1^, ^3^ Physical exam findings may include a left‐sided, parasternal systolic murmur, and if there is significant RVOT, a parasternal heave with a palpable thrill.^1^ An EKG can show findings of RVH, such as right axis deviation and prominent R waves in the right precordial leads.^1^ Echocardiography can be used in diagnosis of DCRV, with continuous Doppler used to determine intraventricular pressure gradients. However, while TTE is usually diagnostic in the pediatric population, it is less accurate compared to TEE in adults. Hoffman et al^5^ found TTE to be diagnostic in only 15.6% of patients, whereas TEE accurately diagnosed all patients studied. Difficulty with TTE is thought to be due to the proximity of the transducer to the outflow tract.^5^

As seen with our patient, DCRV can be difficult to distinguish from TOF as both can present with echocardiographic evidence of a VSD, RVH, overriding aorta and RVOT obstruction. However, there are several notable differences. First, the level of outflow obstruction occurs at anatomically distinct locations. TOF is characterized by obstruction at the infundibulum, the smooth muscular region of the RV outlet, whereas the obstruction in DCRV is subinfundibular, arising from trabeculations. In our patient's case, his cardiac MRI notably showed subpulmonic stenosis. In addition, DCRV is associated with increased pulmonary flow, whereas TOF has decreased pulmonary flow.^1^ If echocardiography is nondiagnostic, cardiac catheterization and MRI can be used.^1^ Treatment involves surgery with myomectomy, especially for patients with an intracavitary systolic pressure gradient >40 mm Hg, aortic regurgitation, or symptoms of heart failure.^1^, ^5^

Few other case reports have described infectious bacterial endocarditis occurring with DCRV.^7^, ^8^ Infective endocarditis is a known complication of CHD and is becoming more prevalent in patients who survive into adulthood.^3^ Several theories exploring the relationship of congenital disease and endocarditis involve blood velocity and hemodynamics. Increased contact between tissue surface area and blood, as seen in valvular congenital anomalies, is thought to favor bacterial localization. Further concepts suggest that endocarditis is seen when blood flows across high‐pressure gradients, such as in ventricular septal defects.^9^ A study of patients with repaired and unrepaired congenital heart anomalies found the most common site of infective endocarditis to be left ventricular outflow lesions. Ventricular septal defect was also a common site of endocarditis in unrepaired CHD. TOF was the most common cyanotic condition associated with endocarditis.^3^

In our case report, we present an adult patient with unrepaired DCRV and VSD presenting with endocarditis. Although endocarditis is an unusual initial presentation for DCRV, patients are at an increased risk due to turbulent blood flow across pressure gradients. Patients with DCRV pose a diagnostic challenge, as this condition is associated with various congenital defects. If echocardiogram is nondiagnostic, cardiac catheterization and cardiac MRI can confirm the diagnosis. This patient was ultimately referred to cardiothoracic surgery for definitive surgical repair.

## CONFLICT OF INTEREST

None declared.

## AUTHOR CONTRIBUTION

NSB: involved in patient care, conceived the idea, drafted manuscript. AS: involved in patient care, assisted with image and figure collection. PS: conceived the idea. DRL: involved in revision. All authors have reviewed final manuscript.
